# Cytokine Hemoadsorption as Rescue Therapy for Critically Ill Patients With SARS-CoV-2 Pneumonia With Severe Respiratory Failure and Hypercytokinemia

**DOI:** 10.3389/fmed.2021.779038

**Published:** 2022-01-10

**Authors:** Juan Carlos Ruiz-Rodríguez, Luis Chiscano-Camón, Adolf Ruiz-Sanmartin, Clara Palmada, Erika Paola Plata-Menchaca, Clara Franco-Jarava, Marcos Pérez-Carrasco, Manuel Hernández-González, Ricard Ferrer

**Affiliations:** ^1^Department of Intensive Care, Vall d'Hebron University Hospital, Vall d'Hebron Barcelona Hospital Campus, Barcelona, Spain; ^2^Shock, Organ Dysfunction and Resuscitation Research Group, Vall d'Hebron Research Institute (VHIR), Vall d'Hebron University Hospital, Vall d'Hebron Barcelona Hospital Campus, Barcelona, Spain; ^3^Departament de Medicina, Universitat Autonoma de Barcelona, Barcelona, Spain; ^4^Department of Immunology, Vall d'Hebron Hospital Universitari, Vall d'Hebron Barcelona Hospital Campus, Barcelona, Spain

**Keywords:** SARS-CoV-2 pneumonia, hemoadsorption, acute respiratory distress syndrome (ARDS), hypercytokinemia, COVID-19

## Abstract

**Introduction:** A dysregulated inflammatory response, known as “cytokine storm”, plays an important role in the pathophysiology of coronavirus 2019 disease (COVID-19). Identifying patients with a dysregulated inflammatory response and at high risk for severe respiratory failure, organ dysfunction, and death is clinically relevant, as they could benefit from the specific therapies, such as cytokine removal by hemoadsorption. This study aimed to evaluate cytokine hemoadsorption as rescue therapy in critically ill patients with SARS-CoV-2 pneumonia, severe respiratory failure refractory to prone positioning, and hypercytokinemia.

**Methods:** In this single center, observational and retrospective study, critically ill patients with SARS-CoV-2 pneumonia, severe acute respiratory failure, and hypercytokinemia were analyzed. All the patients underwent cytokine hemoadsorption using CytoSorb^®^ (Cytosorbents Europe, Berlin, Germany). The indication for treatment was acute respiratory failure, inadequate clinical response to the prone position, and hypercytokinemia.

**Results:** Among a total of 343 patients who were admitted to the intensive care unit (ICU) due to SARS-CoV-2 infection between March 3, 2020 and June 22, 2020, six patients received rescue therapy with cytokine hemoadsorption. All the patients needed invasive mechanical ventilation and prone positioning. A significant difference was found in the pre- and post-treatment D-dimer (17,868 mcg/ml [4,196–45,287] vs. 4,488 mcg/ml [3,166–17,076], *p* = 0.046), C-reactive protein (12.9 mg/dl [10.6] vs. 3.5 mg/dl [2.8], *p* = 0.028), ferritin (1,539 mcg/L [764–27,414] vs. 1,197 ng/ml [524–3,857], *p* = 0.04) and interleukin-6 (17,367 pg/ml [4,539–22,532] vs. 2,403 pg/ml [917–3,724], *p* = 0.043) levels. No significant differences in the pre- and post-treatment interleukin-10 levels (22.3 pg/ml [19.2–191] vs. 5.6 pg/ml [5.2–36.6], *p* = 0.068) were observed. Improvements in oxygenation (prehemoadsorption PaO_2_/FIO_2_ ratio 103 [18.4] vs. posthemoadsorption PaO_2_/FIO_2_ ratio 222 [20.9], *p* = 0.029) and in the organ dysfunction (prehemoadsorption SOFA score 9 [4.75] vs. posthemoadsorption SOFA score 7.7 [5.4], *p* = 0.046) were observed. ICU and in-hospital mortality was 33.7%.

**Conclusions:** In this case series, critically ill patients with COVID-19 with severe acute respiratory failure refractory to prone positioning and hypercytokinemia who received adjuvant treatment with cytokine hemoadsorption showed a significant reduction in IL-6 plasma levels and other inflammatory biomarkers. Improvements in oxygenation and SOFA score were also observed.

## Introduction

Clinicians face several challenges when taking care of patients with coronavirus 2019 disease (COVID-19) (1), as the disease presents three distinct stages of disease progression. Each stage corresponds to the different clinical profiles according to individual responses to therapy and different prognoses (2). These three stages determine the severity of SARS-CoV-2 pneumonia: early, pulmonary, and hyperinflammatory. The hyperinflammatory stage is characterized by a multisystemic inflammatory syndrome, in which serum levels of inflammatory biomarkers increase, resulting in a high risk of organ dysfunction and death (3). The “cytokine storm” plays a central role in the pathophysiology of the disease (4). In the former reports from China, the cytokine storm was recognized as a clinical feature associated with the severity of the clinical condition (5).

As a consequence, the resulting inflammatory response is not homogeneous throughout the course of the disease (6, 7). During the asymptomatic phase, hypercytokinemia is not clinically evident, and, in the subsequent stages, massive cytokine release worsens the clinical course of the disease (8). This progression correlates with the fact that although an important cytokine elevation begins in the first 24 or 48 h of presentation, the clinical hyperinflammatory state becomes evident on days 7–10 from the onset of symptoms. At this stage, clinical deterioration is ubiquitous, and acute respiratory failure occurs progressively. In the lung, hypercytokinemia leads to diffuse alveolar damage, hyaline membrane formation, thrombus formation, fibrin exudates, and fibrotic healing (9), resulting in acute respiratory distress syndrome (ARDS) (10), whose frequency is up to 26% in SARS-CoV-2 infection (11, 12).

However, potentially useful adjuvant treatments do not fit all the patients. Early in the course of COVID-19, avoiding immunosuppression is recommended. In advanced stages, immunomodulation is a cornerstone for treatment interventions. Thus, it is relevant to identify the subgroup of patients who develop a hyperinflammatory response (13), as they could benefit from specific therapies, such as immunomodulation using blood purification strategies (14). Cytokine hemoadsorption therapy could be a promising therapeutic intervention in patients with severe acute respiratory failure (15–17).

This study hypothesizes that cytokine hemoadsorption may improve the hyperinflammatory profile and organ dysfunction in the selected critically ill COVID-19 patients. This study aimed to evaluate cytokine hemoadsorption as rescue therapy in critically ill patients with severe SARS-CoV-2 pneumonia, acute respiratory failure refractory to standard maneuvers, and hypercytokinemia.

## Methods

### Patients and Ethics Approval

In this single center, observational, and retrospective study, critically ill patients with COVID-19 who received cytokine hemoadsorption using CytoSorb^®^ (Cytosorbents Europe, Berlin, Germany) adsorbent, between March 3, 2020 and June 22, 2020 were eligible. All patients were admitted to the ICU of Vall d'Hebron University Hospital, Barcelona, Spain. The study was approved by the local Clinical Research Ethics Committee (PR (AG) 270/2020), and the need for informed consent was waived.

### Inclusion and Exclusion Criteria

The inclusion criteria were the presence of acute respiratory failure (PaO_2_/F_I_O_2_ [arterial oxygen pressure (P_a_O_2_), inspired fraction of oxygen (F_I_O_2_)] ratio <150) with poor response to the prone position, hyperinflammatory state, manifested as interleukin-6 (IL-6) hypercytokinemia (IL-6 > 1,000 pg/ml), and increased levels of ferritin and D-dimer (DD). Poor response to prone positioning was considered when PaO_2_/FiO_2_ ratio remained <150 after the prone position. The exclusion criteria were all patients who did not meet the aforementioned criteria or had a limitation of life-sustaining care, pregnant patients, or patients who had other indications for cytokine hemoadsorption.

### Analyzed Data and Scores

The plasma concentrations of inflammatory biomarkers were analyzed, including IL-6, IL-10, DD, and C-reactive protein (CRP) on ICU admission, immediately before hemoadsorption initiation (prehemoadsorption), and after the procedure (posthemoadsorption). The severity of the disease was evaluated with the Acute Physiology and Chronic Health disease Classification System (APACHE) II (18) and Sequential Organ Failure Assessment (SOFA) scores (19). Both scores were calculated using the worst parameters measured during the first 24 h of admission. Organ dysfunction was assessed by calculating the SOFA score before and after the treatment with hemoadsorption.

Acute respiratory distress syndrome was defined according to the Berlin definition criteria (20). Data on the incidence of acute kidney injury (AKI) or failure, and the need for the continuous renal replacement therapy (CRRT), were collected according to the latest kidney disease: Improving Global Outcomes (KDIGO) Clinical Practice Guideline criteria (21). P_a_O_2_/F_I_O_2_ ratio was calculated before and after each hemoadsorption session. Also, the use of high doses of methylprednisolone (~1.5–2 mg/kg), systemic anticoagulation (22), number of days on mechanical ventilation, duration of ICU stay, and ICU and in-hospital mortality were collected. The study fulfilled the “Strengthening the reporting of observational studies in epidemiology (STROBE)” checklist for the observational studies (23).

### CytoSorb^®^ and Continuous Renal Replacement Therapy

CytoSorb^®^ is a highly bio- and hemocompatible cytokine adsorber approved for use in conditions with increased levels of cytokines. The device is composed of porous polymer beads within a huge and efficient surface area. It allows for adsorption and permanent binding of molecules in the 5–60 kDa range. This range includes the vast majority of cytokines and other inflammatory molecules.

The CytoSorb^®^ filter was connected posthemofilter *via* a close loop circuit to the CRRT pump (Prismaflex, Gambro Lundia AB, Lund, Sweden). CRRT was delivered using the continuous hemodiafiltration mode using an MA 150^®^ hemofilter (Baxter, Illinois, US) at a blood flow rate of 200 ml/min. Anticoagulation was performed with citrate or heparin.

### Measurement of Plasmatic Levels of Cytokines

Plasmatic levels of IL-6 were measured using the automated quantitative immunoassay Cobas^®^ (Roche diagnostics International Ltd, Switzerland), following the instructions of the manufacturer. Circulating levels of IL-10 and soluble CD25 (IL-2Ra) were determined using the microfluidics-based quantitative immunoassay, ELLA^®^ (ProteinSimple, California, US), following the instructions of the manufacturer.

### Statistical Analysis

According to the variable distribution, descriptive data were expressed as mean (SD) or median (interquartile range [IQR], 25–75%). Mann-Whitney U-test was used to compare continuous variables and Fisher's test for categorical variables. All the statistical tests were 2-sided, and a *p*-value < 0.05 was considered statistically significant. An analysis of the required sample size was not performed because of the observational characteristics of the study. Data analysis was conducted using the statistical software package PASW Statistics for Windows, Version 18.0 (SPSS Inc, Chicago, Illinois, US).

## Results

### Characteristics of the Study Population

Among a total of 343 patients who were admitted to the ICU due to severe infection of SARS-CoV-2, six patients received treatment with CytoSorb^®^ (Table 1). During the study period, hemoadsorption was performed in another patient whose indication was refractory septic shock secondary to the intestinal perforation. This patient was excluded from this study.

**Table 1:** Clinical patient characteristics.

**Variable**	**Result**
**Characteristics of the study population**
Male gender	5 (83.0%)
Age (years)	57.0 (10.5)
APACHE II	19.5 (6.1)
Body mass index	29 (3)
**Comorbidities**
History of smoking (*n*, %)	2 (33.3%)
Arterial Hypertension (*n*, %)	3 (50%)
Diabetes mellitus (*n*, %)	2 (33.3%)
Chronic Obstructive Pulmonary Disease (*n*, %)	0 (0%)
Malignant condition or immunosuppression (*n*, %)	0 (0%)
Chronic kidney disease (*n*, %)	0 (0%)
Liver disease (*n*, %)	0 (0%)
Congestive heart disease (*n*, %)	0 (0%)
Coronary heart disease (*n*, %)	0 (0%)
**Organ dysfunction and supportive treatment**
SOFA ICU admission	5.2 (1.5)
PaO_2_/F_I_O_2_ ratio	97.5 (14.6)
Prone position (*n*, %)	6 (100%)
Sepsis (*n*, %)	0 (0%)
Shock (*n*, %)	1 (16.7%)
AKI (*n*, %)	3 (50.0%)
CRRT (*n*, %)	3 (50.0%)
Tocilizumab (*n*, %)	4 (66.7%)
Corticoesteroids 2 mg/kg (*n*, %)	1 (16,7%)
Anticoagulation (*n*, %)	4 (66.7%)
VAP (*n*, %)	2/6 (33.3%)
**Hemoadsorption**
Duration hemoadsorption (h)	16.0 (9.0)
N° sessions hemoadsorption	1.2 (1.0)
**Inflamatory parameters on admission**
DD (n.v. <0.5mcg/ml)	559 (254–2643)
CRP (n.v. <0.5mg/dl)	19.5 (13.4)
Ferritin (n.v. <336 mcg/L)	967 (682–2116)
IL-6 (n.v. <4.3 pg/ml)	1163 (52–2775)
**Outcomes**
Days on mechanical ventilation	15.2 (7.0)
ICU stay (days)	17.2 (8.0)
ICU Mortality (*n*, %)	2 (33.3%)
Inhospital Mortality (*n*, %)	2 (33.3%)

*AKI, acute kidney injury; APACHE II, Acute Physiology and Chronic Health disease Classification System II; CRRT, continuous renal replacement therapy; CRP, C-reactive protein; DD, D-dimer; ICU, intensive care unit; SOFA, sequential organ failure assessment; VAP, ventilator-associated pneumonia*.

From a total of six patients, 5 (83.0%) were male; mean age 57.0 years (10.5). Patient characteristics, other treatments received, and outcomes are presented in Table 1. All the patients fulfilled the Berlin criteria for severe ARDS. All patients required mechanical ventilation and prone positioning. The mean SOFA score was 5.2 (1.5) at ICU admission, and the mean P_a_O_2_/F_i_O_2_ ratio was 97.5 (14.6). The levels of DD were 559 mcg/ml (254–2,643), CRP 19.5 mg/dl (13.4), ferritin 967 mcg/L (682–2,116) and IL-6 1,163 pg/ml (52–2,775).

The mean duration of the mechanical ventilation was 15.2 days (7.2). Three (50%) patients developed COVID-19-associated AKI and required CRRT. The mean ICU stay was 17.2 days (8.0), and ICU and in-hospital mortality was 33.7%.

### Hemoadsorption

Patient eligibility for the cytokine hemoadsorption was assessed between days 3 and 4 of ICU admission. At inclusion, clinical parameters of the organ dysfunction and inflammation had worsened in all patients. The mean SOFA score was 9 (4.75), and P_a_O_2_/F_i_O_2_ ratio was 103 (18.4). Levels of DD were 17,868 mcg/ml (4,196–45,287), CPR 12.9 mg/dl (10.6), ferritin 1,539 mcg/L (764–27,414), IL-6 17,367 pg/ml (4,539–22,532), and IL-10 22.3 pg/ml (19.2–191) (Table 2). All the patients underwent cytokine hemoadsorption (5 patients received one session of CytoSorb^®^ hemoadsorption [each session of variable duration], and one patient received two 24-h sessions). The mean perfusion time was 16 (9) h. The circuit patency determined the duration of hemoadsorption sessions in 3 patients (circuit clotting occurred at 3, 8, and 16 h).

**Table 2:** Comparison of prehemoadsorption (pre-HA) and posthemoadsorption (post-HA) parameters.

	**ICU admission**	**Pre-HA**	**Post-HA**	***p*-value**
SOFA	5.2 (1.5)	9 (4.75)	7.7 (5.4)	*p* = 0.046
PaO_2_/F_I_O_2_ ratio	97.5 (14.6)	103 (18.4)	222 (20.9)	*p* = 0.029
DD (n.v. <0.5 mcg/ml)	559 (254–2,644)	17,868 (4,196–45,287)	4,488(3,166–17,076)	*p* = 0.046
CRP (n.v. <0.5 mg/dl)	19.5 (13.4)	12.9 (10.6)	3.5 (2.8)	*p* = 0.028
Ferritin (n.v. <336 mcg/L)	967 (682–2,116)	1,539 (764–27,414)	1,197 (524–3,857)	*p* = 0.046
IL-6 (n.v. <4.3 pg/ml)	1,163 (52–2,775)	17,367 (4,539–22,532)	2,403 (917–3,724)	*p* = 0.043
IL-10 (n.v. <7.8 pg/ml)	–	22.3 (19.2–191)	5.6 (5.2–36.6)	*p* = 0.068

*DD, D-dimer; Fb, fibrinogen; HA, hemoadsorption; IL-6, interleukin 6; IL-10, interleukin 10; CRP, C-reactive protein; n.v., normal values; SOFA, Sequential Organ Failure Assessment score*.

### Inflammatory Parameters and Organ Dysfunction

All inflammatory parameters, except for IL-10 levels, significantly decreased after treatment (posthemoadsorption DD levels 4,488 mcg/ml [3,166–17,076], *p* = 0.046; posthemoadsorption CRP 3.5 mg/dl [2.8], *p* = 0.028; posthemoadsorption ferritin levels 1,197 mcg/L [524–3,857], *p* = 0.046; posthemoadsorption IL-6 levels 2.403 pg/ml [917–3.724], *p* = 0.043; and posthemoadsorption IL-10 levels 5.6 pg/ml [5.2–36.6], *p* = 0.068).

Improvements in oxygenation (posthemoadsorption P_a_O_2_/F_i_O_2_ ratio 222 (20.9), *p* = 0.029) and the organ dysfunction were also observed (posthemoadsorption SOFA score 7.7 [5.4], *p* = 0.046) (Table 2).

## Discussion

### Main Findings of This Study

This retrospective study describes the potential benefits of CytoSorb^®^ hemoadsorption in critically ill patients with refractory acute respiratory failure due to COVID-19 and hypercytokinemia. Hemoadsorption was associated with a reduction in inflammatory biomarkers, improved oxygenation, and multiorgan dysfunction.

### Previous Experience With Cytokine Hemoadsorption

Several recommendations regarding the use of cytokine hemoadsorption in SARS-CoV-2 pneumonia have been published recently. The National Health Commission and National Administration of Traditional Chinese Medicine recommends CytoSorb^®^ hemoadsorption to treat severe and critical cases of COVID-19 (24). The Brescia Renal Covid Task Force recommends CytoSorb^®^ hemoadsorption in the patients with COVID-19 admitted to ICU who have ARDS or AKI-requiring CRRT (25). The Panamanian Association of Critical Medicine and Intensive Therapy recommends using CytoSorb^®^ in patients with hyperlactatemia and high-dose vasopressors who do not respond to standard therapy. Patients with severe ARDS with high-ventilatory support requirements or candidates for the use of extracorporeal membrane oxygenation (ECMO) therapy are also considered (26). The Colombian consensus suggests using CytoSorb^®^ in patients with cytokine storm syndrome when there is a lack of treatment response, and while evaluating the individual prognosis of the patient (27). On April 10, 2020, the United States of America Food and Drug Administration issued an Emergency Use Authorization for CytoSorb^®^ to treat patients with 18 years of age or older with confirmed COVID-19 admitted to the ICU with confirmed or imminent respiratory failure and specifically early ARDS, severe disease or life-threatening disease defined as respiratory failure, septic shock or the multiorgan dysfunction (28). Despite these recommendations, clinical experience is scarce and comes mainly from case reports and some case series (29–31).

### Characteristics of the Study Population. Target Patient Population

The selection of patients with COVID-19 for receiving cytokine hemoadsorption is critical and should be individualized. There are two clinical phenotypes of the patients with COVID-19 (11). One phenotype is characterized by a mild or moderate disease with low-viral loads. These patients have preserved interferon responses with regulated production of cytokines and show rapid recovery from initial lymphopenia. Thus, they are unlikely to benefit from cytokine hemoadsorption. However, selected patients in the second phenotype, characterized by a severe disease with a high risk of death, high-viral loads, insufficient interferon response, sustained lymphopenia, and a very significant elevation of cytokines, could benefit from cytokine hemoadsorption. In this study, patients were more suitable to receive cytokine hemoadsorption if they were more severely ill and developed severe acute respiratory failure refractory or poorly responsive to prone positioning, in association with a hyperinflammatory state (determined by very high levels of biomarkers, such as IL-6, ferritin, and DD). All patients in this study presented a considerable deleterious clinical condition, with a mean PaO_2_/F_I_O_2_ ratio of 103 (18.4).

There is a lack of studies evaluating cytokine hemadsorption in critically ill patients with COVID-19, and some of them have included heterogeneous populations of critically ill patients. Rampino et al. (32) reported a case series of 9 consecutive critically ill patients with SARS-CoV-2 and acute respiratory failure requiring continuous positive airway pressure. In this study, no patients required invasive mechanical ventilation. Their eligibility criteria were confirmed SARS-CoV-2 pneumonia and a sum of P_a_O_2_/F_i_O_2_ ratio <200 mm Hg, CRP levels >10 mg/dl, and a lymphocyte count <1,500/mm^3^. Damiani et al. (33) delivered hemoadsorption with CytoSorb^®^ for 24 to 48-h sessions to 11 patients with COVID-19 requiring mechanical ventilation due to rapidly progressive ARDS after a median of 3 days (range 0–4 days) from hospital admission. Nassiri et al. (34) used CytoSorb^®^ in 26 patients with COVID-19-associated moderate ARDS (P_a_O_2_/F_i_O_2_ ratio <200) and hyper inflammation (CRP > 50 mg/L and ferritin > 1,500 mcg/L). Of all patients, 46.2% received mechanical ventilation. Paisey et al. (35) reported a case series of 15 patients with severe COVID-19 that received cytokine hemoadsorption (five HA-330 cartridges and 10 CytoSorb^®^ adsorbents). All the patients needed invasive mechanical ventilation and CRRT, and 11 received ECMO support. In a multicenter study, Villa et al. (36) evaluated 37 patients who had received cytokine hemoadsorption using the oXiris^®^ membrane. The indication for oXiris^®^ was biochemical and clinical evidence of systemic inflammation associated with AKI, hemodynamic instability, or multiorgan dysfunction. All the patients received mechanical ventilation.

### Biomarker Levels and Organ Dysfunction Throughout Hemoadsorption in Relation to Outcomes

In general, critically ill patients with COVID-19 do not show increased plasma levels of biomarkers as other populations of critically ill patients (e.g., septic shock or sepsis with patients with ARDS). Previous studies have found mild-to-moderate elevations of CRP, IL-6, and ferritin. (37) However, there are no well-defined thresholds of biomarkers to consider the initiation of cytokine hemoadsorption. Given the heterogeneity of individual responses and the numerous underlying factors that affect levels of biomarker, it is uncertain whether valid thresholds will be determined shortly.

The results of this study are engaging and coincide with the previous studies. Rampino et al. (32) documented reductions in proinflammatory cytokines (e.g., IL-6) in patients receiving cytokine hemoadsorption therapy. All patients who received the treatment survived, and only two of them needed endotracheal intubation. Damiani et al. (33) showed the median values of IL-6 before hemoadsorption were 355 pg/ml (IQR 263–466), 118 pg/ml (IQR 19–221, *p* = 0.003) at treatment end and 169 pg/ml (IQR 61–253, *p* = 0.03) 24 h after therapy. A significant decrease in CRP and an increase in P_a_O_2_/F_i_O_2_ ratio were also observed. The improvement in the inflammatory profile was associated with progressive improvements in the respiratory function. Nassiri et al. (34) reported that the P_a_O_2_/F_i_O_2_ ratio, SOFA score, and inflammatory biomarkers (procalcitonin, CRP, and ferritin) improved significantly, and the authors reported a mortality rate of 19.2%. A potential limitation of this study is that cytokine levels were not reported. Paisey et al. (35) proposed hemoadsorption as an adjunctive treatment leading to a reduction in ferritin, CRP, procalcitonin, and lactate levels. Yet, no significant differences were found in IL-6 and IL-10 pre- and post-treatment levels. In these patients, hypercytokinemia was moderate, although they showed a hyperinflammatory profile based on ferritin and CPR levels. Villa et al. (36) delivered the hemoadsorption therapy after a median of 3.6 days (IQR 3.7) from ICU admission and 14 days (IQR 10.0) from the symptom onset. The decrease of IL-6 concentration was significant, especially during the first 24 h of treatment (from baseline levels of 1,230 pg/ml [IQR 895] to 479 pg/ml [IQR 531] at 24 h after treatment, 320 pg/ml [IQR 259] at 48 h, and 160 pg/ml [IQR 141] at 72 h [*p* = 0.001 for each time point]). The reduction in serum IL-6 concentration levels correlated with improved organ function, particularly hemodynamic and pulmonary function. A slight decrease in the observed mortality rate compared with the predicted mortality rate, calculated by the APACHE IV score, was also observed.

The results of this study are different from the recently published CYCOV trial (38), in which 34 patients with severe COVID-19 pneumonia requiring ECMO were included. Seventeen patients were treated with cytokine hemoadsorption for 72 h. No significant differences in IL-6 levels were observed between the two groups after 72 h of cytokine hemoadsorption. The median IL-6 concentrations decreased from 357 pg/ml (IQR 177.4–118.0) to 98.6 pg/ml (71–192.8) in the cytokine adsorption group and from 289.0 pg/ml (87–787.0) to 110.0 pg/ml (48–198.5) in the control group. In contrast, the median baseline IL-6 levels were very high (17,367 pg/ml [4,539–22,532]), and the reduction in IL-6 levels and inflammatory biomarkers was substantial. The results of the CYVOV trial are not comparable with the results of this study, as the rate of cytokine removal by hemoadsorption depends on the presence of baseline high-concentration levels of cytokines in plasma (39).

In this study, the strategy for delivering cytokine hemadsorption was different from other studies that used fixed hemoadsorption regimens. Single 24-h sessions of hemoadsorption were planned to be delivered (only one patient required two sessions of 24 h). Real-time IL-6 levels were monitored during the hemoadsorption sessions, which allowed us to withhold the treatment at 24 h if IL-6 levels had been reduced, and the patient had clinical improvement. As observed by Damiani et al. (33) and Paisey et al. (37), no significant differences were found in the reduction of IL-10 levels. However, the lack of effect over IL-10 levels may be secondary to the slightly increased baseline levels and the small sample size.

Cytokine hemoadsorption could be considered an effective and safe rescue therapy for highly selected critically ill patients with COVID-19. Further studies and randomized controlled trials in critically ill patients with COVID-19 with refractory acute respiratory failure and hypercytokinemia should be conducted to accurately narrow the indications and clinical benefits of the cytokine hemoadsorption.

### Limitations

This study has some limitations. First, this is a single-center study including a small number of patients with no control group. Thus, the findings of this study should be confirmed in larger comparative studies and cannot be extrapolated to other ICU settings; moreover, the complexity of the conferred difficulty of the patients in determining the effect of CytoSorb^®^ alone on patient outcomes. Second, the patient inclusion process was not consecutive. Given the unprecedented pandemic situation, it was impossible to ensure that all patients meeting the inclusion criteria were evaluated for eligibility. Third, we have not performed the sample size calculation because of the observational characteristics of the study.

## Conclusions

In this case series, critically ill patients with COVID-19 with severe acute respiratory failure refractory to prone positioning and hypercytokinemia who received adjuvant treatment with cytokine hemoadsorption showed a significant reduction in IL-6 plasma levels and other inflammatory biomarkers. Improvements in oxygenation and SOFA score were also observed. Cytokine hemoadsorption could be a safe and effective rescue therapy for patients with refractory COVID-19 acute respiratory failure.

## Data Availability Statement

The original contributions presented in the study are included in the article/supplementary materials, further inquiries can be directed to the corresponding author/s.

## Ethics Statement

The study was approved by the Local Clinical Research Ethics Committee (PR (AG) 270/2020), and the need for informed consent was waived. The patients/participants provided their written informed consent to participate in this study.

## Author Contributions

All authors involved in providing care for the patient and writing and reviewing the manuscript.

## Conflict of Interest

The authors declare that the research was conducted in the absence of any commercial or financial relationships that could be construed as a potential conflict of interest.

## Publisher's Note

All claims expressed in this article are solely those of the authors and do not necessarily represent those of their affiliated organizations, or those of the publisher, the editors and the reviewers. Any product that may be evaluated in this article, or claim that may be made by its manufacturer, is not guaranteed or endorsed by the publisher.
